# Patients Receiving 100 or More Electroconvulsive Therapy Treatments in a Nationwide Cohort

**DOI:** 10.1111/acps.70137

**Published:** 2026-08-26

**Authors:** Masahiro Hata, Yuki Miyazaki, Shun Takahashi

**Affiliations:** ^1^ Department of Psychiatry Osaka University Graduate School of Medicine Osaka Japan; ^2^ Kosaka Hospital Osaka Japan; ^3^ Clinical Research and Education Center Asakayama General Hospital Osaka Japan; ^4^ Department of Occupational Therapy, Graduate School of Rehabilitation Science Osaka Metropolitan University Osaka Japan; ^5^ Department of Neuropsychiatry Wakayama Medical University Wakayama Japan

**Keywords:** claims database, clozapine, cumulative exposure, electroconvulsive therapy, real‐world evidence

## Abstract

**Introduction:**

Very high cumulative exposure to electroconvulsive therapy (ECT) has primarily been described in individual case reports. We examined cumulative ECT exposure in a nationwide Japanese claims database to characterize patients receiving very high numbers of treatments.

**Methods:**

We identified 3,733 patients who received ECT and classified them according to cumulative ECT exposure. Clinical characteristics of patients receiving 100 or more treatments were examined.

**Results:**

Among 3,733 patients who received ECT, 39 (1.0%) received 100 or more treatments. These patients had treatment durations of several years and substantially greater numbers of psychiatric hospitalizations and cumulative hospitalization days. Clozapine use increased across cumulative ECT exposure categories, although it was recorded in only 13% of patients receiving 100 or more treatments.

**Conclusion:**

Patients receiving 100 or more ECT treatments represent an identifiable subgroup of ECT recipients characterized by prolonged and recurrent psychiatric care.

## Key Points

- Significant outcomes
  ○ In this nationwide cohort, 1% of electroconvulsive therapy recipients received 100 or more treatments, representing an identifiable subgroup of electroconvulsive therapy recipients.
  ○ Patients receiving 100 or more treatments had treatment durations of several years and substantially more psychiatric hospitalizations and cumulative hospitalization days, indicating prolonged and recurrent psychiatric care.
  ○ Clozapine use increased across cumulative electroconvulsive therapy exposure categories, although the clinical significance of this association could not be determined from claims data.
- Limitations
  ○ Treatment intent could not be determined from claims data; therefore, acute, continuation, and maintenance electroconvulsive therapy could not be reliably distinguished.
  ○ Hospital‐specific patient identifiers prevented linkage across institutions, potentially resulting in duplicate patient records and incomplete ascertainment of cumulative electroconvulsive therapy exposure.
  ○ Claims data lacked detailed clinical information, including treatment response and cognitive outcomes, limiting assessment of the long‐term tolerability and clinical context of repeated electroconvulsive therapy.

## 1 Introduction

Electroconvulsive therapy (ECT) may be continued for years in patients with severe and recurrent psychiatric illness. Although individual case reports have described patients receiving 100 or more ECT treatments over prolonged periods [1, 2, 3], the frequency and clinical characteristics of such patients remain unknown. Population‐level data on these patients may provide insights into long‐term ECT practice. We therefore examined the frequency and clinical characteristics of patients receiving 100 or more ECT treatments in a nationwide cohort.

### 1.1 Aims of the Study

This study aimed to determine the frequency and clinical characteristics of patients receiving 100 or more electroconvulsive therapy treatments in a nationwide cohort. We also examined their psychiatric hospitalization burden and clozapine use in relation to cumulative treatment exposure.

## 2 Methods

We conducted a retrospective cohort study using a deidentified nationwide hospital claims database provided by Medical Data Vision Co. Ltd, including demographic, diagnosis, procedure, prescription, and hospitalization data from acute care hospitals participating in Japan's Diagnosis Procedure Combination system. The database captures more than half of ECT procedures performed in Japan [4]. We identified all patients receiving ECT between April 2008 and March 2025 and calculated cumulative ECT exposure from procedure records. The study was approved by the Osaka University Hospital Ethics Committee (approval No. 25119), which waived informed consent because deidentified data were used.

Patients were categorized by cumulative ECT exposure as fewer than 20, 20 to 49, 50 to 99, or 100 or more treatments; 100 ECT treatments was selected as a pragmatic threshold for identifying patients with high cumulative ECT exposure. We extracted age, sex, ICD‐10 F2 (schizophrenia‐spectrum disorders) and F3 (mood disorders) diagnoses, ECT treatment duration, psychiatric hospitalizations, cumulative hospitalization days, and clozapine exposure. Diagnostic categories were determined using diagnosis recorded on or before the date of the first ECT treatment.

Characteristics were compared between patients receiving fewer than 100 and 100 or more ECT treatments using the Mann–Whitney *U* test and Fisher exact test. The odds of clozapine use across cumulative ECT exposure categories were evaluated using logistic regression, with cumulative ECT exposure treated as an ordinal variable and adjustment for age at first ECT and sex. Tests were two‐sided, with *p* < 0.05 indicating statistical significance. Analyses were performed using Python (version 3.9.6).

## 3 Results

Among 3733 ECT recipients, 3056 (81.9%) received fewer than 20 treatments, 526 (14.1%) received 20 to 49, 112 (3.0%) received 50 to 99, and 39 (1.0%) received 100 or more treatments (Table 1). Patients receiving 100 or more treatments had longer ECT treatment duration (median, 2405 vs. 30 days), more psychiatric hospitalizations (median, 38 vs. 1), and more cumulative hospitalization days (median, 673 vs. 106 days) than those receiving fewer than 100 treatments. Age, sex, and diagnostic composition did not differ significantly between groups.

**TABLE 1:** Characteristics of patients by cumulative electroconvulsive therapy exposure.

Characteristic	< 20 (*n* = 3056)	20–49 (*n* = 526)	50–99 (*n* = 112)	≥ 100 (*n* = 39)	*p* value, ≥ 100 versus < 100
Age, median (IQR), years	59 (44–71)	62 (47–72)	62 (52–72)	65 (54–71)	0.12
Female sex, *n* (%)	1998 (65.4)	368 (70.0)	82 (73.2)	30 (77)	0.18
ECT treatment duration, median (IQR), days	27 (18–38)	512 (194–1320)	1555 (982–2338)	2405 (1820–3198)	< 0.001
Psychiatric admissions, median (IQR)	1 (1–2)	3 (2–5)	11 (6–23)	38 (16–66)	< 0.001
Cumulative psychiatric hospitalization days, median (IQR), days	88 (49–171)	211 (138–356)	402 (291–584)	673 (486–867)	< 0.001
F2 only, *n* (%)	774 (25.3)	128 (24.3)	28 (25.0)	10 (25.6)	1
F3 only, *n* (%)	407 (13.3)	52 (9.9)	12 (10.7)	3 (7.7)	0.471
Both F2 and F3, *n* (%)	1867 (61.1)	344 (65.4)	72 (64.3)	26 (66.7)	0.621
Clozapine use, *n* (%)	89 (2.9)	30 (5.7)	7 (6.3)	5 (13)	0.01

*Note:* Age was defined as age at the first ECT treatment. The total number of hospitalizations and cumulative hospitalization days represent all psychiatric hospitalizations recorded throughout the study period (April 2008 to March 2025), irrespective of whether they occurred before or after the initiation of ECT. Ten patients without recorded ICD‐10 F2 or F3 diagnoses on or before the first ECT were excluded from the diagnostic category analyses. The *p* values compare patients receiving fewer than 100 and 100 or more ECT treatments. F2 and F3 indicate ICD‐10 schizophrenia‐spectrum disorders and mood disorders, respectively.

Abbreviations: ECT, electroconvulsive therapy; IQR, interquartile range.

Clozapine use increased across cumulative ECT exposure categories, from 89 of 3056 patients (2.9%) receiving fewer than 20 treatments to 5 of 39 (13%) receiving 100 or more treatments. The diagnostic composition was broadly similar across cumulative ECT exposure categories (Table 1). After adjustment for age at first ECT and sex, the odds of clozapine use increased with increasing cumulative ECT exposure (adjusted OR per category increase, 1.92; 95% CI, 1.52–2.44; *p* < 0.001), with patients receiving 100 or more treatments having greater odds of clozapine use than those receiving fewer than 100 treatments (adjusted OR, 6.04; 95% CI, 2.23–16.36; *p* < 0.001); however, 34 of 39 patients (87%) had no recorded clozapine use.

## 4 Discussion

In this nationwide cohort, 1% of ECT recipients received 100 or more treatments. Continuation and maintenance ECT are valuable treatment approaches for preventing relapse and recurrence following a successful acute course of ECT, particularly in patients with severe and recurrent illness [5]. Treatment should be individualized according to each patient's clinical needs, preferences, treatment response, and risk of adverse effects [6]. In Japan, nationwide information on patients receiving prolonged continuation or maintenance ECT remains limited. Against this background, our nationwide cohort provides population‐level data on patients receiving high cumulative numbers of ECT treatments, a population for whom only limited evidence has previously been available [1, 2, 3]. Our findings show that these patients represent an identifiable subgroup of ECT recipients.

Patients receiving high cumulative numbers of ECT treatments may be familiar to clinicians at individual institutions, yet their broader epidemiologic presence has remained largely unrecognized. Because such patients are uncommon, individual institutions may encounter only one or a few cases, which may be regarded as unusual cases shaped by local clinical circumstances. By aggregating ECT practice across institutions, our findings place these seemingly isolated cases into a broader population context.

Patients receiving 100 or more ECT treatments had treatment durations of several years and substantially more psychiatric hospitalizations and cumulative hospitalization days. These findings indicate that high cumulative ECT exposure is associated with prolonged and recurrent psychiatric care. Clozapine use also increased across cumulative ECT exposure categories, suggesting that patients with greater cumulative ECT exposure were more likely to have received clozapine. However, clozapine use was identified in only 13% of patients receiving 100 or more treatments. The relatively low proportion of clozapine use should be interpreted cautiously because it may reflect diagnostic heterogeneity, contraindications, patient preference, prescribing practices, health care system factors, or diagnostic indications beyond schizophrenia‐spectrum disorders, none of which could be evaluated using claims data.

This study has limitations. Because the claims database does not contain information on treatment intent, we could not reliably distinguish acute, continuation, and maintenance ECT, nor determine the number of patients receiving maintenance ECT. In addition, because patient identifiers are hospital‐specific in the MDV database, patients transferred between hospitals could not be linked. Consequently, duplicate patient records and incomplete ascertainment of cumulative ECT exposure may have occurred. Claims data lacked information on ECT indications, symptom severity, treatment response, and reasons for clozapine nonuse. Therefore, the long‐term tolerability profile associated with cumulative ECT exposure, including cognitive outcomes, could not be evaluated. Diagnoses may have been subject to misclassification, and clozapine use is an imperfect marker of treatment resistance, particularly in Japan, where its use remains relatively limited [7]. The findings may not generalize to health care systems with different ECT utilization rates, clinical practices, and clozapine prescribing patterns. Nevertheless, this nationwide study shows that patients receiving 100 or more ECT treatments are not limited to isolated case reports but represent an identifiable subgroup of ECT recipients.

## Author Contributions

Masahiro Hata had full access to all the data in the study and takes responsibility for the integrity of the data and the accuracy of the data analysis. Concept and design: Masahiro Hata. Acquisition, analysis, or interpretation of data: All authors. Drafting of the manuscript: Masahiro Hata. Critical revision of the manuscript for important intellectual content: All authors. Statistical analysis: Yuki Miyazaki. Supervision: Shun Takahashi.

## Funding

This study was partially supported by JSPS KAKENHI (grant JP25K10813).

## Conflicts of Interest

The authors declare no conflicts of interest.

## Data Availability

The data that support the findings of this study are available from Medical Data Vision Co. Ltd (MDV). Restrictions apply to the availability of these data, which were used under license for this study. Data are available from https://en.mdv.co.jp/ with the permission of Medical Data Vision Co. Ltd (MDV).
